# Clonality and geographic structure of host-specialized populations of *Corynespora cassiicola* causing emerging target spot epidemics in the southeastern United States

**DOI:** 10.1371/journal.pone.0205849

**Published:** 2018-10-15

**Authors:** Leilani G. Sumabat, Robert C. Kemerait, Dong Kyun Kim, Yeshwant R. Mehta, Marin T. Brewer

**Affiliations:** 1 Department of Plant Pathology, University of Georgia, Athens, Georgia, United States of America; 2 Department of Plant Pathology, University of Georgia, Tifton, Georgia, United States of America; 3 Instituto Agronômico do Paraná-IAPAR, Londrina, Brazil; University of Sydney, AUSTRALIA

## Abstract

*Corynespora cassiicola* is a destructive plant-pathogenic fungus causing widespread target spot epidemics, including outbreaks on cotton, soybean, and tomato in the southeastern United States. Previous studies revealed that populations from the three hosts are genetically distinct and host specialized. Although variation in aggressiveness to cotton and tomato were observed, no genetic diversity was detected within populations sampled from each of these hosts. We aimed to gain a better understanding of the emerging target spot epidemics by developing microsatellite markers for *C*. *cassiicola* to assess genetic variation, population structure, and to infer modes of reproduction and mechanisms of dispersal. Two hundred sixty-five isolates from cotton, soybean, tomato, and other host plants were genotyped with 13 microsatellite markers. Genotypic diversity revealed genetic variation within each of the populations collected from different hosts, with the population from cotton dominated by clonal genotypes and showing the least genetic diversity. In addition, *C*. *cassiicola* populations on different host species were genetically distinct and structured based on host species. No association between genetic and geographic distances was identified in the tomato populations, and the association in cotton populations was low. However, significant regional geographic structure was detected in the soybean populations of *C*. *cassiicola*. These results further support previous findings of introduced host specialized isolates or the evolution of more aggressive strains on each host. The lack of geographic structure suggests that the clones on cotton and tomato spread rapidly, or similar founder populations were established by human-mediated dispersal, and that dispersal is not limited. However, regional geographic structure of populations on soybean suggests limited dispersal among more established populations of *C*. *cassiicola*, or genetic differences in founder populations that colonized different geographic areas.

## Introduction

Asexual reproduction is common among many fungal and oomycete plant pathogens, resulting in clonal populations with parents and offspring that are genetically identical [1]. Although strictly clonal organisms lack the capacity to overcome deleterious mutations, many of the known destructive plant pathogens predominantly reproduce asexually [2]. Previous studies have documented the evolutionary potential and epidemiological implications of plant pathogens with clonal populations, and oftentimes, emerging and re-emerging plant diseases are caused by clonal lineages resulting from pathogen introductions, host jumps, or geographic range expansions [3–5]. A few of these are classic examples of plant pathogens that substantially impacted agriculture or forest ecosystems. These include: *Phytophthora infestans*, which causes late blight of tomato and potato [6], where epidemics in Europe and United States were identified as introductions of clonal lineages carrying either A1 or A2 mating types [7]; *Phytophthora ramorum*, which causes the widespread outbreaks of sudden oak death in the Pacific Northwest forests, where studies on the genetic diversity and reproductive mode revealed that clonal lineage NA1 dominates in the region and no evidence of sexual reproduction has been detected [8]; and *Cryphonectria parasitica*, which causes chestnut blight, where studies have provided evidence for clonal expansion in Europe [9].

*Corynespora casssiicola* (Berk & Curtis) Wei is causing target spot epidemics on cotton (*Gossypium hirsutum*) [10–15], soybean (*Glycine max*) [16–18], and tomato (*Solanum lycopersicum*) [19] in the southeastern U.S. Moreover, target spot epidemics on both cotton and soybean have become severe in Brazil [20] where these crops are also widely grown. The phylogenetic diversity and host specialization of *C*. *cassiicola* collected from a variety of crop, ornamental, and weed species worldwide has been described [21, 22]. Six well-supported clusters, referred as phylogenetic lineages (PL), were identified based on four loci (ITS, *act1*, *caa5*, and *ga4*). The PL were associated with the host of origin and pathogenicity but were widely distributed geographically. Phylogenetic analyses of *C*. *cassiicola* particularly from cotton, tomato, and soybean in the southeastern U.S. showed three genetically distinct populations that clustered based on the host species of origin, with isolates from cotton and soybean belonging to PL1 and isolates from tomato belonging to PL4 [21]. Furthermore, isolates were most aggressive when inoculated onto the same host as the host of origin, indicating evidence of host specialization. Interestingly, the populations specialized to both cotton and tomato showed no genetic diversity among the four sequenced loci, although variation in virulence was detected. Larger sample sizes and more variable markers are needed to detect variation within the populations, to determine if the populations are clonal, and to investigate geographic structure within populations.

Determining the spatial genetic structure of emerging pathogen populations assists in understanding how pathogens spread and helps in identifying sources of inoculum [23–24]. Inferences on the dispersal patterns of the pathogens can be made by comparing the genetic distances and geographic distances of individuals from the same epidemics [25]. A lack of genetic similarity relative to geographic proximity resulting in no spatial genetic structure indicates long-distance movement, whereas significant correlation implies local or limited dispersal [24]. In *C*. *cassiicola*, conidia have been observed in the field serving as survival structures on plant debris and as the source of inoculum for primary as well as secondary infections during the growing season [26]. However, genetic similarity of populations over geographic regions and long-distance dispersal mechanisms of *C*. *cassiicola* are not yet known.

The life cycle of *C*. *cassiicola*, which belongs to the phylum Ascomycota, class Dothideomycetes, order Pleosporales [27] is considered strictly asexual, because sexual structures have never been observed. Most studies have shown clonal lineages in *C*. *cassiicola* resulting from asexual reproduction [22, 28–30]. Sumabat et al. [21] showed evidence of recombination among the lineages from different hosts in the southeastern U.S.; however, this recombination was likely historical or due to homoplasy since the reticulations were detected deep within the lineages. Fungi previously thought to be strictly asexual due to lack of observed sexual reproductive structures have later shown evidence for sexual reproduction including the presence of both mating types and/or genetic recombination [1, 31].

Population genetic analyses can improve our ability to understand emerging epidemics [2, 32]. Patterns of variation can be identified and used to infer evolutionary processes. In addition, population genetic analyses can be used to infer sources of inoculum, pathogen dispersal mechanisms, reproductive modes and mating systems, and population genetic structure. Genetic markers are needed to detect genetic variation among individuals. Microsatellite markers had been informative in detecting diversity within populations of the same species [23, 33–35]. These markers are also referred to as simple sequence repeats (SSR), and consist of one to six nucleotides that are repeating in tandem and are located throughout the genomes. They are ideal for many population genetic studies, especially for clonal organisms with limited genetic diversity, because they are highly polymorphic, co-dominant, species-specific, easy to amplify through PCR, and selectively neutral [36]. In addition, they are relatively inexpensive when multiplexed [37].

Our goal was to gain a better understanding of the emergence of the target spot epidemics on cotton, soybean, and tomato. This included developing polymorphic microsatellite markers for *C*. *cassiicola* and genotyping a much larger sample of isolates from a wider geographic scale, including isolates from cotton and soybean target spot epidemics in Brazil. We were interested in determining if epidemics in the U.S. could be caused by similar populations to those causing epidemics in Brazil. Specifically, we wanted to assess the genetic variation within host specialized populations of *C*. *cassiicola* to determine if they are clonal reproducing, and if they are geographically structured or if dispersal is widespread.

## Materials and methods

### Sampling and DNA extraction

Two hundred sixty-five isolates of *C*. *cassiicola* were collected from infected leaves of cotton, soybean, tomato, cucumber, hydrangea, *Mandevilla*, pepper, and sesame and fruits of tomato from the southeastern U.S. (Table 1). The required APHIS PPQ permit for interstate movement of plant pathogens was acquired. Isolates were obtained from a single target spot lesion per leaf or fruit. An approximately 1-cm^2^ of the lesion was surface sterilized in 0.6% NaOCl for 1 min, rinsed twice with sterile distilled water, and blotted dry with a low-lint wipe. The surface-sterilized lesion was divided into 4 sections that were placed onto a 100-mm Petri dish containing quarter-strength potato dextrose agar (qPDA). After 3 days of incubation in the dark at 25°C, an agar plug containing mycelia from the edge of one of the colonies was transferred to a new dish of qPDA. All isolates were processed for DNA extraction using a rapid, high yield mini-prep method for fungi [38]. Prior to extraction, agar plugs of pure cultured isolates were transferred to qPDA overlaid with sterile cellophane in 60 x 15 mm Petri dishes. The isolates were grown in the dark for 5 days at 25°C. Newly grown hyphae were then carefully scraped from the surface of the cellophane with a sterile spatula and added to 1 mL of lysis buffer (0.5M EDTA [pH 8], 1M Tris [pH 8], 20% sodium dodecyl sulfate, proteinase K at 20 mg/mL, and 1% sodium bisulfite), vortexed for approximately 1 min and incubated at 65°C for 15 min. Each sample was then briefly vortexed again and centrifuged at 12,500 rpm for 5 min. The resulting supernatant was transferred to a new, sterile tube and 200 μL of 7.5M NH_4_OAc was added, vortexed on high for approximately 10 seconds, placed on ice for 15 min, and then centrifuged at 12,500 rpm for 3 min. The supernatant was transferred to another sterile tube, 700 μL isopropanol was added, it was thoroughly mixed, and then centrifuged at 12,500 rpm for 5 min. Once completed, the supernatant was carefully poured off. The remaining pelleted DNA at the bottom of the tube was rinsed twice with 1 mL of 70% EtOH, dried, and re-suspended with 50 μL of sterile H_2_O. DNA was stored at -20°C. Genomic DNA was obtained from cotton and soybean isolates in Brazil.

**Table 1:** Origin of *Corynespora cassiicola* isolates used in this study.

Host	Location	Year	Isolate ID (*N*)
Cotton (*Gossypium hirsutum*)	Mitchell Co., GA, USA	2013	CM1, CM3, CM4, CM5, CM7, CM8, CM9, CM10, CM11, CM12, CM13^1^^,^ ^2^^,^ ^3^, CM14, CM15, CM16, CM17, CM18, CM19, CM20 (18)
Cotton	Mitchell Co., GA, USA	2015	CTP1, CTP2, CTP3, CTP4, CTP7, CTP9 (6)
Cotton	Tift Co., GA, USA	2013	CT1^3^, CT2, CT3, CT4, CT2-1, CT2-2, CT2-3, CT2-4 (8)
Cotton	Tift Co., GA, USA	2015	CGA-2, CGA-3, CGA-4, CGA-5, CGA-6, CGA-7, CGA-8, CGA-9, CGA-10 (9)
Cotton	Pierce Co., GA, USA	2013	CPi1^3^, CPi2, CPi3, CPi1-1, CPi2-1, CPi2-2, CPi2-3, CPi2-4, CPi3-1, CPi3-2, CPi4-1, CPi4-2, CPi4-3, CPi4-4, CPi5-2, CPi6-2, CPi7-1, CPi7-2, CPi7-3, CPi7-4, CPi8-1^3^, CPi8-2, CPi8-5, CPi9-1, CPi9-2 (25)
Cotton	Coffee Co., GA, USA	2013	CCo-1, CCo-2, CCo-3, CCo-4 (4)
Cotton	Ware Co., GA, USA	2013	CW1, CW5 (2)
Cotton	Atkinson Co., GA, USA	2013	CA1, CA4 (2)
Cotton	Bishop Co., GA, USA	2014	CB1, CB2, CB3 (3)
Cotton	Miller Co., GA, USA	2015	CTGA_m1, CTGA_m2, CTGA_m3, CTGA_m4, CTGA_m5, CTGA_m7, CTGA_m8, CTGA_m9, CTGA_m10, CTGA_m1a, CTGA_m3a, CTGA_m9a, CTGA_m10a (13)
Cotton	Thomas Co., GA, USA	2015	TCU1, TCU2, TCU3, TCU4, TCU5, TCU7, TCU8, TCU9, TCU10, TCUa1, TCUa4, TCUa5, TCUa6, TCUa7, TCUa8, TCUa9, TCUa10, TCT2, TCT3, TCT8 (20)
Cotton	Seminole Co., GA, USA	2015	CTGA_S1-1, CTGA_S1-8, CTGA_S2-2, CTGA_S2-5, CTGA_S2-10 (5)
Cotton	Duval Co., FL, USA	2013	FlM1, FlM2, FlM3, FlM4 (4)
Cotton	Madison Co., TN, USA	2013	CTs1^3^, CTs2, CTs3, CTs4 (4)
Cotton	Madison Co., TN, USA	2014	CTN2a-1^3^, CTN2a-2, CTN2a-3, CTN2a-4, CTN2a-5, CTN2b-1, CTN2b-2, CTN2b-4, CTN2b-5, CTN2c-1, CTN2c-2, CTN2c-3, CTN2c-4, CTN2c-5, CTN2d-1, CTN2d-4, CTN2d-5 (17)
Cotton	Gibson Co., TN, USA	2014	CTNa-1^3^, CTNa-2, CTNa-3, CTNa-4, CTNa-5, CTNb-1, CTNb-3, CTNb-5, CTNc-1 (9)
Cotton	Suffolk Co., VA, USA	2013	CVa1, CVa2, CVa3, CVa4, CVa5^1^^,^ ^2^^,^ ^3^ (5)
Cotton	Henry Co., AL, USA	2015	CAL-1^3^ (1)
Cotton	Baldwin Co., AL, USA	2015	CAL-2, CAL-2a (2)
Cotton	Elmore Co., AL, USA	2015	CAL-3 (1)
Cotton	Macon Co., AL, USA	2015	CAL-4 (1)
Cotton	Rapides Co., LA, USA	2014	CLAa1, CLAa2, CLAb2, CLAb3, CLAb5, CLAc2, CLAc4, CLAc5 (8)
Cotton	Craighead Co., AR, USA	2015	CARa-3, CARa-4, CARa-6, CARa-7, CARa-10 (5)
Cotton	Mississippi Co., AR, USA	2015	CARb-1, CARb-2, CARb-3, CARb-4, CARb-5, CARb-6, CARb-7, CARb-9, CARb-10 (9)
Cotton	Matto Grosso, Brazil	2016	5CCA, 7CCA, 9CCA, 10CCA (4)
Soybean (*Glycine max*)	Tift Co., GA, USA	2013	SSTa1^2^^,^ ^3^, SSTa2, SSTa3, SSTa4, SSTa5, SSTb3, SSTb4, SSTb5 (8)
Soybean	Tift Co., GA, USA	2014	SGa2, SGa4 (2)
Soybean	Marion Co., GA	2013	SMR1, SMR2, SMR3, SMR4 (4)
Soybean	Suffolk Co., VA, USA	2013	SVa1 (1)
Soybean	Madison Co., TN, USA	2013	STs1, STs2 (2)
		2014	STNa-1^3^, STNa-2, STNa-3, STNa-5, STNb-1, STNb-2, STNb-3, STNb-4, STNb-5, STNc-3, STNc-4, STNc-5, STNd-3 (13)
Soybean	Calhoun Co., GA, USA	2013	GaT-S3 (1)
Soybean	Poinsett Co., AR, USA	2015	SAR2, SAR4, SAR7, SAR8, SAR9, SAR10, SAR11, SAR13 (8)
Soybean	Matto Grosso, Brazil	2016	3CCS, 4CCS (2)
*Hydrangea* sp.	Tift Co., GA, USA	2013	HT1, HT3, HT4, HT5, HT6, HT8, HT9, HT14 (8)
*Hydrangea* sp.	Tift Co., GA, USA	2014	HGa1, HGa2, HGa3, HGa4 (4)
*Hydrangea* sp.	Oconee Co., GA, USA	2013	GaA-H1^3^, GaA-H2^3^ (2)
*Hydrangea* sp.	Newton Co., GA, USA	2013	GaN-H2, GaN-H3 (2)
Tomato (*Solanum lycopersicum*)	Cairo Co., GA, USA	2013	TCf2^4^, TCl1, TCl2, TCl3^2^^,^ ^3^, TCl4, TCl5 (6)
Tomato	Hillsborough Co., FL, USA	2014	7p, 108, 1343, 1551 (4)
Tomato	Collier Co., FL, USA	2014	1555^3^ (1)
Pepper (*Capsicum annuum*)	Echols Co., GA, USA	2013	PE1, PE2, PE3, PE4, PE5 (5)
Pepper	Tift Co., GA, USA	2013	PE2-1, PE2-2, PE2-3, PE2-4 (4)
*Mandevilla* sp.	Oconee Co., GA, USA	2013	GaA-Hb1 (1)
Cucumber (*Cucumis sativus*)	Colquitt Co., GA, USA	2013	CuC1 (1)
Sesame (*Sesamum indicum*)	Tift Co., GA, USA	2014	SeF1 (1)

^1^Isolates sequenced for whole genome and assembled to search for microsatellite repeats

^2^Isolates used to test for PCR amplification of the designed primers

^3^Panel of isolates used to test for polymorphisms

^4^ Isolate from tomato fruit

### Development of microsatellite markers and genotyping of isolates

Microsatellite markers for *C*. *cassiicola* were developed through a next generation sequencing approach. High quality genomic DNA was extracted from two *C*. *cassiicola* isolates from cotton, CM13 and CVa5 (Table 1), using the cetyl trimethylammonium bromide (CTAB) method [39]. Isolates were grown on a 100-mm Petri dish of qPDA overlaid with sterile cellophane and incubated in the dark at 25°C for 7 days. A total of 17.5 ml of CTAB lysis buffer was added to approximately 500 mg of mycelium finely ground in liquid nitrogen. The CTAB buffer is composed of the following: 6.5 ml of Buffer A (0.35 M sorbitol; 0.1 M Tris–HCl, pH 9; and 5 mM EDTA, pH 8), 6.5 ml of Buffer B (0.2 M Tris–HCl, pH 9; 50 mM EDTA, pH 8; 2 M NaCl; 2% CTAB), 2.6 ml of Buffer C (5% Sarkosyl), 1.75 ml PVP (0.1%), and 1.25 μl Proteinase K. The mixture was shaken with 2 5-mm glass beads (VWR Soda Lime, Radnor, PA, USA) at 1750 RPM for 2 min using a 2010 Geno/Grinder (SPEX SamplePrep, Metuchen, NJ, USA). 5.75 ml 5 M potassium acetate was then added in the tube, inverted ten times and incubated on ice for 30 min. These were then centrifuged for 20 min at 14, 000 *g*. The resulting supernatant was added to one volume of chloroform-isoamylalcohol (v/v 24:1) and then centrifuged at 14, 000 *g* for 10 min. The supernatant was then added to 100 μl RNase A (10 mg/ml) and incubated at 37°C for 120 min. Isopropanol at equal volume and sodium acetate at 1/10 volume were then added and incubated at 25°C for 5 min. These were centrifuged at 14, 000 *g* for 30 min and the supernatant was discarded. The resulting pellet was rinsed twice with 70% ethanol and air-dried overnight. The DNA pellet was subsequently dissolved in 100 μl deionized H_2_O. Genomic DNA was submitted to the Georgia Genomics and Bioinformatics Core (GGBC–Athens, GA, USA) for library preparation of each isolate and Illumina sequencing using a NextSeq platform based on a paired-end 150-bp (PE150) protocol.

To identify microsatellite loci shared among isolates of *C*. *cassiicola* from diverse hosts, yet polymorphic within cotton isolates, the forward and reverse reads of CM13 and CVa5 were trimmed and mapped to a *C*. *cassiicola* reference genome (Corca1, NCBI project ID 234811) [40] from a rubber (*Hevea brasiliensis*) isolate using default settings in Geneious v.6 (Biomatters). Using Phobos 3.3.11 [41] we identified contigs with trimeric or tetrameric microsatellite sequences at least 15 nucleotides in length within each of the draft genome assemblies. The contigs with microsatellites from CVa5 were mapped to the contigs from CM13 using Geneious. The microsatellite loci were scanned for regions that were polymorphic between the cotton isolates.

Primers flanking repeat regions that were polymorphic for the two cotton isolates were designed in Primer3 [42]. Each forward primer was tagged with a CAG-tag sequence at the 5' end (5'-CAGTCGGGCGTCATCA) for use with a labelled fluorescent primer in a three-primer reaction [43] in downstream genotyping analyses. A pigtail sequence (5'-GTTTCTT) [44] was added at the 5' end of the reverse primers to avoid stuttering. The sets of primers for 32 microsatellite loci were tested for PCR amplification on four *C*. *cassiicola* isolates, including CVa5 and CM13 from cotton, as well as Ssta1 from soybean, and TCl3 from tomato. PCR amplification was performed in a 10-μl reaction volume containing 1 μl of 10–200 ng/μl genomic DNA template, 1 μl of 10× PCR buffer (Takara Bio, Inc.), 1 μl of 2.5 mM dNTPs, 0.5 μl of 10 μM forward primer, 0.5 μl of 10 μM reverse primer, and 0.1 μl ExTaq (Takara Bio, Inc.). Thermal cycling conditions had an initial denaturation for 5 min at 95°C followed by; 28 cycles of 30 s at 95°C, 90 s at 57°C, and 30 s at 72°C; and a final elongation of 30 min at 60°C. Amplification of a product was confirmed by electrophoresis on a 1% (w/v) agarose gel with 1× TBE buffer.

PCR primers that produced single products of the expected size for the four isolates in the initial screen were further tested for polymorphism on a panel of 15 *C*. *cassiicola* isolates representative of different hosts and geographic regions (Table 1). The three-primer method [43] was used in this stage of marker selection to reduce the cost of fluorescent primers. The three primers for each marker included a forward primer with a CAG-tag sequence at the 5' end, the reverse primer, and a CAG-tag primer labeled at the 5' end with the fluorescent dye HEX (Integrated DNA Technologies). PCR amplification was performed in a 12.5 μl reaction containing the following: 1 μl of 10–200 ng/μl genomic DNA template; 1.2 μl of 10× PCR buffer (Takara Bio, Inc.), 1.2 μl of 2.5 mM dNTPs; 0.05 μl of 10 μM 5' CAG-tag labeled forward primer; 0.5 μl of 10 μM reverse primer; 0.5 μl of 10μM 5' HEX-labeled CAG-tag primer; and 0.5 U of Taq polymerase (Takara Bio, Inc.). Thermal cycling conditions and confirmation of PCR products by gel electrophoresis were conducted as described above. PCR products were diluted 1:15 in double-distilled H_2_O, then 1 μl of diluted PCR product was combined with 0.1 μl of the internal size standard Genescan-500 Liz (Applied Biosystems) and 9.9 μl of Hi-Di formamide (Applied Biosystems). Samples were incubated at 95°C for 5 min and placed immediately on ice. Fragment analysis was conducted at the GGBC on an Applied Biosystems 3730x1 96-capillary DNA Analyzer. Microsatellite Plugin in Geneious v.6 (Biomatters) was used to score alleles and loci were distinguished based on expected size range.

Multiplex PCR was developed for cost-effective and high-throughput genotyping of *C*. *cassiicola*. Primers that produced single peaks in the expected size range and amplified markers that were polymorphic among the panel of fifteen diverse isolates (Table 1) were selected for two multiplex reactions (Table 2). The forward primers were labeled at the 5' end with one of the following fluorescent dyes: 6FAM, VIC, PET, or NED (Applied Biosystems). Multiplex reactions were designed so that markers with allele sizes within a similar range were labelled with different fluorescent dyes. The same panel of fifteen isolates was used to optimize two sets of multiplex reactions.

**Table 2:** Repeat unit, primer sequences, multiplexing primers and reactions, size and number of observed alleles, and estimated gene diversity for *C*. *cassiicola* microsatellite loci.

Locus	Repeat motif	Primer sequence (5' to 3')^1^	Fluor-escent label for F Primer	Multiplex reaction	Range of Allele Sizes (bp)	No. of Obs. alleles	Gene diversity^2^ Simpson Nei	
Cc4_1	(ACAG)_5_	F: GGAGCCGTACCAAGCCTC R: GGCACTACTACATGGGACGA	PET	1	237–245	3	0.26	0.26
Cc4_2	(ATC)_5_	F: ATCTCCCTCCCACATTGTCC R: TGGTTGGGTGTTCTGCAATG	PET	1	298–313	5	0.24	0.25
Cc10_3	(ACC)_5_	F: CCAGCCAAAAACTCTGCACC R: AACCAAGAGGGCCAAGATGG	NED	1	270–287	7	0.42	0.42
Cc11_2	(ATC)_5_	F: AACGGAACTACCCCAACGAC R: CAGGAAATTTTTGGGGCGCA	6FAM	1	330–346	6	0.49	0.50
Cc12_2	(AAC)_13_	F: CCAACAACCTAACGGTCCGA R: CAATGCAGGCATCGACGATG	6FAM	1	229–314	15	0.54	0.54
Cc12_3	(ACCT)_4_	F: GCTGTTACTGTTGCTGCTGG R: TCCTCCACCCACTACTACCG	6FAM	2	279–297	15	0.61	0.62
Cc14_2	(AGCC)_6_	F: TTTCCGTTATCAGACCGGGC R: GCTTCTAATGCCGGCCATTG	PET	2	246–273	10	0.41	0.42
Cc14_3	(ATC)_7_	F: ATCAAAATGGAGAGGGCGCA R: TTGAACCTTGGGGGACAACTG	VIC	2	289–335	11	0.45	0.45
Cc15_2	(GGC)_5_	F: GGCGGGGAAAAATTGGGAAC R: GGTGTTGGTGCTGAGTCAGA	6FAM	2	355–358	4	0.32	0.32
Cc19_2	(ACTC)_8_	F: TATTGCTGCGTCATCTGCCT R: CGTTCCCCTTGAATGCTTGC	NED	2	289–300	7	0.48	0.49
Cc20_1	(ACAG)_3_	F: GTTGGCTGGCTGCTTGTTTG R: ACGCTAGAAACCGTCCAGTC	6FAM	2	214–243	12	0.51	0.51
Cc25_1	(CTT)_7_	F: ATCAATGAGGCGGTGAGGAG R: CTCGACGCTCACTATCCCAC	6FAM	1	226–265	10	0.51	0.51
Cc26_1	(GAT)_5_	F: ACAGTCGTCGACAGAACACC R: CCCTAGCGTCCTGTTGACTC	VIC	1	285–295	6	0.26	0.26
Mean						8.54	0.42	0.42

^1^ F primers were labeled at the 5'end with the one of the fluorescent dyes (6FAM–Integrated DNA Technologies; VIC, PET, or NED–Applied Biosystem) and all R primers have a GTTTCTT at the 5'end

^2^ Simpson’s index and Nei’s 1978 gene diversity

Multiplex PCR was performed using the Type-it Microsatellite PCR Kit (Qiagen) following the manufacturer’s instructions, except reactions were scaled to 10 μl. Multiplex reactions consisted of 5 μl of 2× Type-it Master Mix, 1 μl of 10× primer mix (consisting of 2 μM of each primer in the multiplex, except Cc12_2 and Cc15_2 which were 3 μM each), 1 μl of DNA template, and RNAse-free water. Thermal cycling conditions were the same as described earlier. PCR products were diluted 1:15 in double-distilled H_2_O and 1 μl of diluted PCR product was combined with 0.1 μl of the internal size standard Genescan-500 Liz (Applied Biosystems) and 9.9 μl of Hi-Di formamide (Applied Biosystems). Samples were incubated at 95°C for 5 min and placed immediately on ice. Fragment analysis was conducted as described above. Microsatellite Plugin in Geneious v.7 was used to score alleles and loci were distinguished based on fluorescent dye and expected size range. The capability of the microsatellite loci to resolve genotypes was determined by plotting the proportion of multilocus genotypes against the number of loci analyzed with 1,000 randomizations of the data in poppr package in R.

### Population genetic analyses

Measures of allelic and genotypic diversity, clonal composition, and population genetic structure, as well as relationships among haplotypes from different host populations were based on the multilocus genotype data generated by the developed microsatellite markers. The number of observed alleles and gene diversity for each locus were estimated using the poppr package performed in R [45]. Gene diversity was based on Simpson and Nei’s indices to account for richness indicating abundance of alleles and evenness indicating distribution or rarity of alleles for each microsatellite locus. The number of unique multilocus genotypes (*g*) was estimated in MLGsim 2.0 [46]. Genotypic diversity (*Ĝ*), which is the probability that two individuals taken at random will have unique multilocus genotypes, was estimated for the overall population that included all 265 isolates and for populations from each host in MultiLocus v.1.3b [47]. The total number of repeated, or clonal, genotypes, and the corresponding number of individuals in each clonal genotype were determined in MLGsim 2.0. Shannon-Weaver’s evenness index (*H*) was estimated in poppr package in R [45].

To visualize patterns of genetic variation and determine if populations are structured, discriminant analysis of principal components (DAPC) in R [48] and principal coordinates analysis (PCoA) in GenAlEx v.6.5 [49] were conducted. DAPC is a multivariate analysis that is independent of any evolutionary models. Clusters were identified through *K*-means clustering of principal components, where *K* is the optimal number of clusters and is inferred as the number of clusters where the Bayesian information criterion (BIC) increases or decreases by a negligible amount. PCoA was generated based on pairwise genetic distances of genotypes of all isolates. The resulting principal coordinate values generated for PC1 and PC2 for each isolate in each population (cotton, soybean, hydrangea, and the tomato/pepper/cucumber/mandevilla population) were compared using one-way analysis of variation (ANOVA). To examine relationships among all isolates and isolates from tomato, cotton, and soybean populations, minimum spanning networks were constructed using the Bruvo’s genetic distance model [50] in the Poppr package executed in R [45].

To identify regional geographic genetic structure and provide insight on the modes of pathogen dispersal during the epidemics, a *Mantel Test* [51] was performed in GenAlEx v.6.5 [49] by comparing matrices of standardized pairwise genetic distances (Φ_*PT*_/(1−Φ_*PT*_)) to account for sample size errors and non-parametric distribution and the logarithm of geographic distance.

## Results

### Microsatellite marker development

A total of 227 and 224 scaffolds were obtained for the CM13 and CVa5 draft genomes, respectively, when mapped to the 244 scaffolds of the reference genome, Corca1 [40]. Of these scaffolds, 62 and 64 contained microsatellite repeats with the criteria of trimeric or tetrameric microsatellite sequences at least 15 nucleotides in length. The scaffolds with microsatellite regions were aligned and 31 polymorphic microsatellite loci were identified on separate scaffolds. Although additional polymorphic regions were likely present, we began marker optimization at this point. Nineteen of the 31 primer pairs produced a single visible band for all 4 *C*. *cassiicola* isolates from cotton, tomato, and soybean when tested for PCR amplification and electrophoresed on a gel.

On a panel of 15 *C*. *cassiicola* isolates from different hosts and geographic origins in the southeastern U.S., 14 of the 19 markers that amplified were reproducible and polymorphic. Two multiplex reactions were developed for high throughput genotyping of isolates. Each reaction contained 7 primer pairs that varied in allele sizes or fluorescent dye label, and were optimized by slightly modifying the concentrations of primers of loci with inconsistent amplification or low peaks. One marker was removed because when mixed with other primer pairs it did not amplify consistently or produce scoreable alleles, even after attempts at optimization. Thus, 13 microsatellite markers (Table 2) were used to genotype the 265 *C*. *cassiicola* isolates. However, no peaks were produced for 95%, 95%, and 82% of the tomato isolates for the markers Cc4_2, Cc15_2, and Cc26_1 regions, respectively, in the multiplex reactions despite multiple attempts to optimize the conditions, including modification of DNA concentrations and primers and thermal cycling conditions. Therefore, we genotyped most of the tomato isolates for these three markers using single marker PCR, which worked well when amplified singly.

The observed number of alleles varied from 3 to 15 for each locus with a mean of 8.5 alleles per locus. Measures of gene diversity ranged from 0.24 to 0.61 and 0.25 to 0.62 for Simpson’s index and Nei’s gene diversity, respectively, showing different levels of richness and evenness across loci. Average gene diversity (0.42 for both Simpson’s index and Nei’s gene diversity) was relatively high, especially for a haploid system.

**Fig 1:**
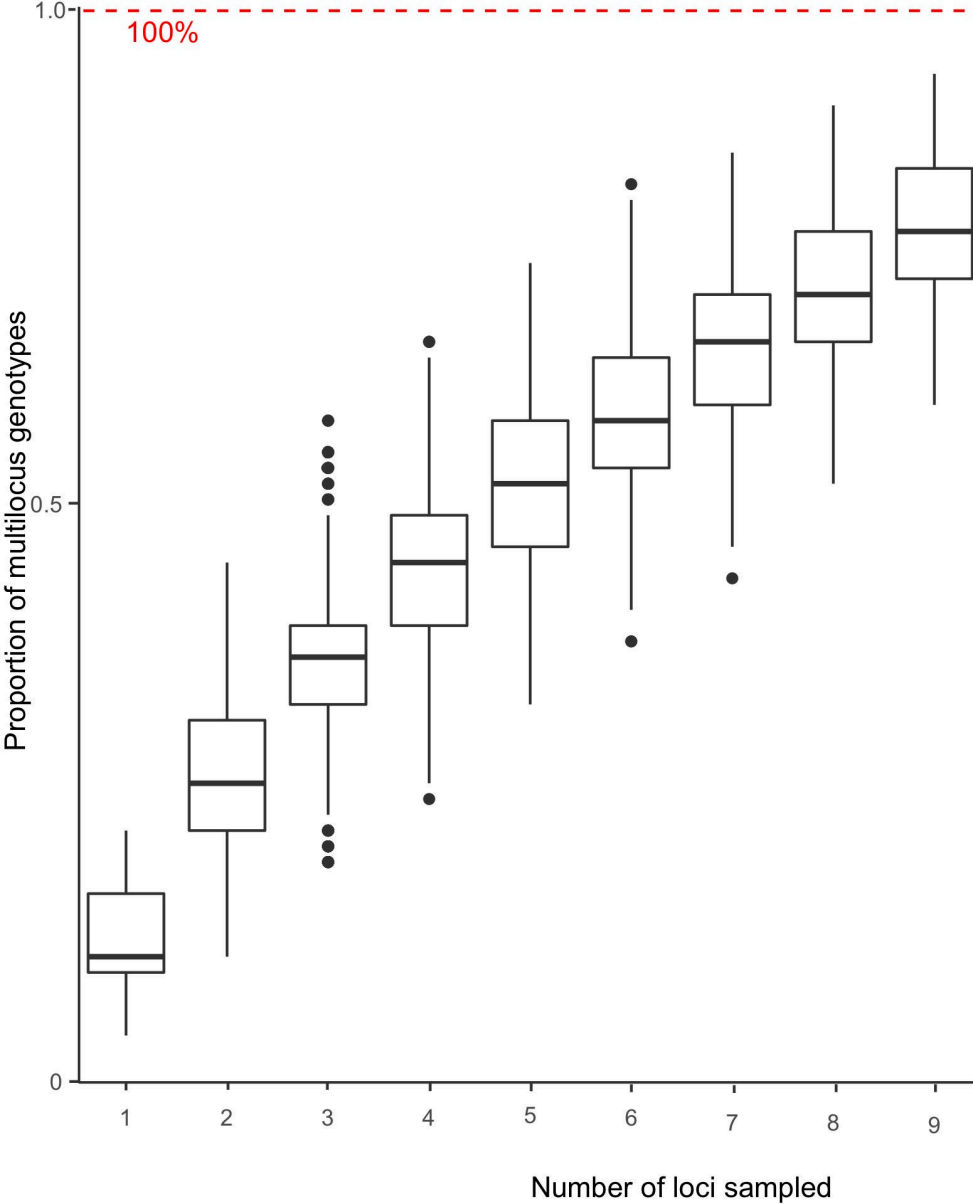
Genotype accumulation curve for *C*. *cassiicola*. Proportion of multilocus genotypes identified based on the number of loci sampled. There were 1,000 randomizations of the data analyzed in poppr package executed in R [45].

To determine the sufficient number of microsatellite loci that can distinguish genotypes, we plotted the proportion of genotypes and the number of loci sampled (Fig 1). Five loci can identify over 50% of the multilocus genotypes and 12 loci nearly identify 100% of the multilocus genotypes.

### Genetic diversity and clonal composition

Of the 265 *C*. *cassiicola* isolates genotyped with 13 microsatellite markers, we detected 71 unique multilocus genotypes with genotypic diversity of 0.79 for all isolates (Table 3). Among the different hosts in the southeastern U.S., *C*. *cassiicola* populations from tomato and soybean have higher genotypic diversity of 0.83 and 0.86, respectively, whereas the population from cotton showed the least genotypic diversity at 0.56. Numerous clonal genotypes, identified as multilocus genotypes with two or more individuals, were detected within the *C*. *cassiicola* populations (Table 3). Overall, 73% of the *C*. *cassiicola* isolates were clonally-related, with 17 multilocus genotypes repeated across 2 to 112 individuals. The population from cotton showed the highest clonal composition at 88%, whereas the population from tomato showed the lowest clonal composition at 36%. Despite the detection of numerous clones representing a majority of the overall population, they were repeated within host populations and none of the clones occurred across populations from cotton, soybean, tomato or hydrangea. The three isolates from cotton in Brazil shared the same genotype with clones from soybean in the U.S., whereas one isolate from cotton and 2 isolates from soybean in Brazil shared the same genotype with a different clone group of soybean isolates from the U.S.

**Table 3:** Genotypic diversity and clonal composition of *Corynespora cassiicola* populations in the southeastern U.S. based on the host of origin.

Population	*N*^*1*^	*g*^*2*^	*Ĝ*^*3*^	*H*^*4*^	Clonal genotypes^5^	Number of individuals in each clonal genotype
Overall^6^	265	71	0.79	2.77	17	112, 20, 15, 11, 11, 9, 5, 4, 4, 4, 3, 3, 2, 2, 2, 2, 2
Cotton	181	22	0.56	1.51	6	112, 20, 15, 11, 4, 3
Soybean	39	23	0.83	2.74	5	11, 4, 2, 2, 2
Tomato^7^	22	14	0.86	2.41	3	5,4,2
Hydrangea	16	7	0.68	1.45	2	9,2

^1^ Total number of individuals

^2^ Number of multi-locus genotypes

^3^ Genotypic diversity

^4^ Shannon-Weaver’s index

^5^ Number of genotypes represented by two or more individuals

^6^ Overall population, including isolates from Brazil and sesame

^7^ The tomato population also includes isolates from pepper, cucumber, and mandevilla since they shared genotypes in the current study and in a previous study [21].

### Population genetic structure

**Fig 2:**
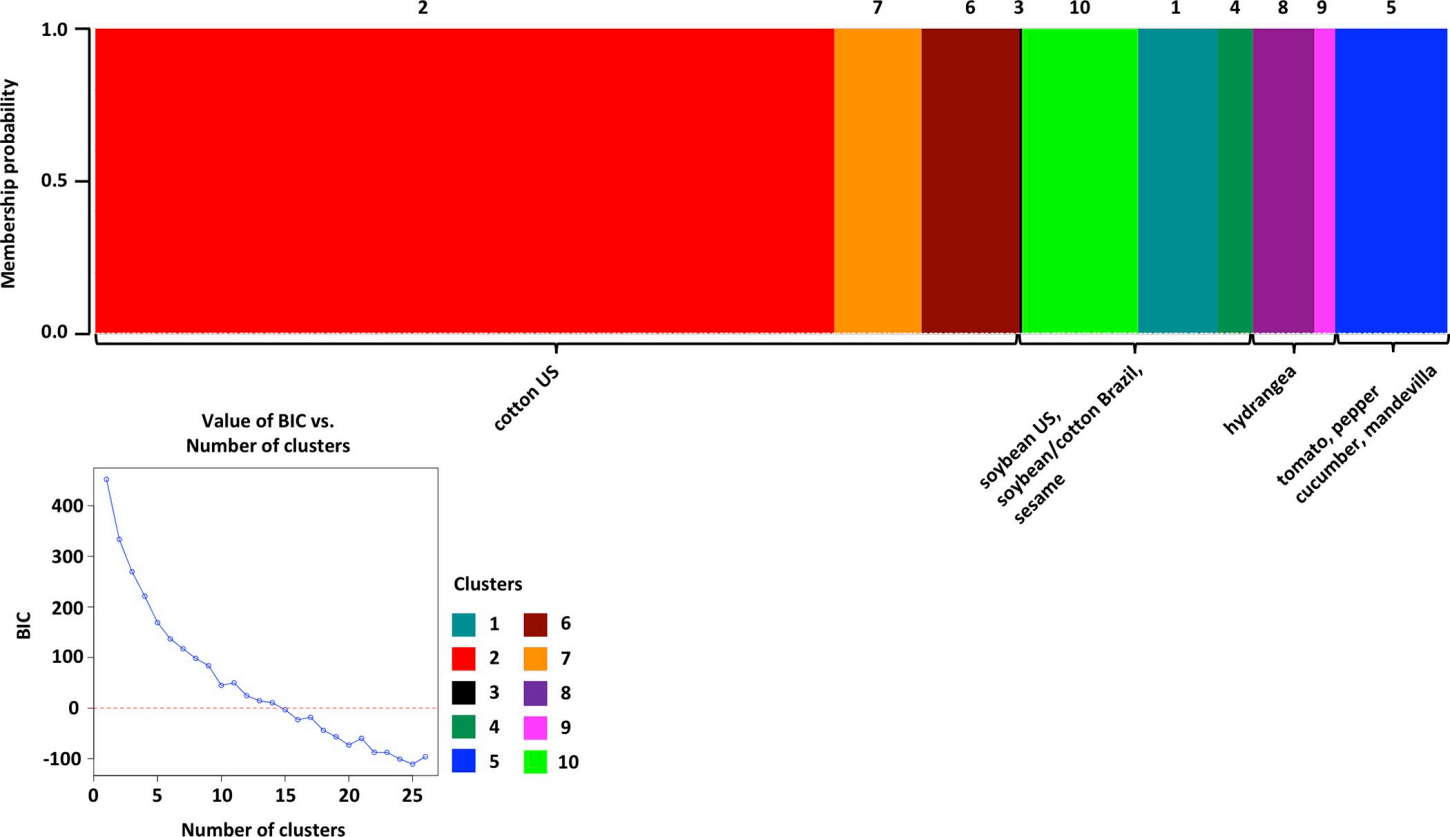
Histogram of assignment probability of 265 *Corynespora cassiicola* isolates to clusters based on discriminant analysis of principal components (DAPC). Each bar represents a single isolate and the color shown indicates the predicted cluster composition in proportion to the bar size. Additionally, numbers are above the histogram to indicate each cluster. The Bayesian information criterion (BIC) for each K value is shown in the lower left corner.

Population genetic structure of *C*. *cassiicola* was characterized by discriminant analysis of principal components (DAPC) and principal coordinates analysis (PCoA) of the genotype data. For DAPC, K = 10 optimal clusters were identified (Fig 2). Each of the isolates had 100% assignment probability to a cluster. The majority of the clusters were represented by clonal genotypes from the host populations in the southeastern U.S. or Brazil. Cotton isolates from the southeastern U.S. were distributed among clusters 2, 6, and 7, with isolates from the large clone group with 112 members belonging to cluster 2. Soybean isolates from the southeastern U.S. were distributed among clusters 1, 3, 4, and 10, with cluster 1 also containing cotton isolates from Brazil and cluster 4 containing the isolate from sesame and cotton and soybean isolates from Brazil. Tomato, pepper, mandevilla, and cucumber isolates belonged to cluster 5, and hydrangea isolates belonged to clusters 8 and 9.

**Fig 3:**
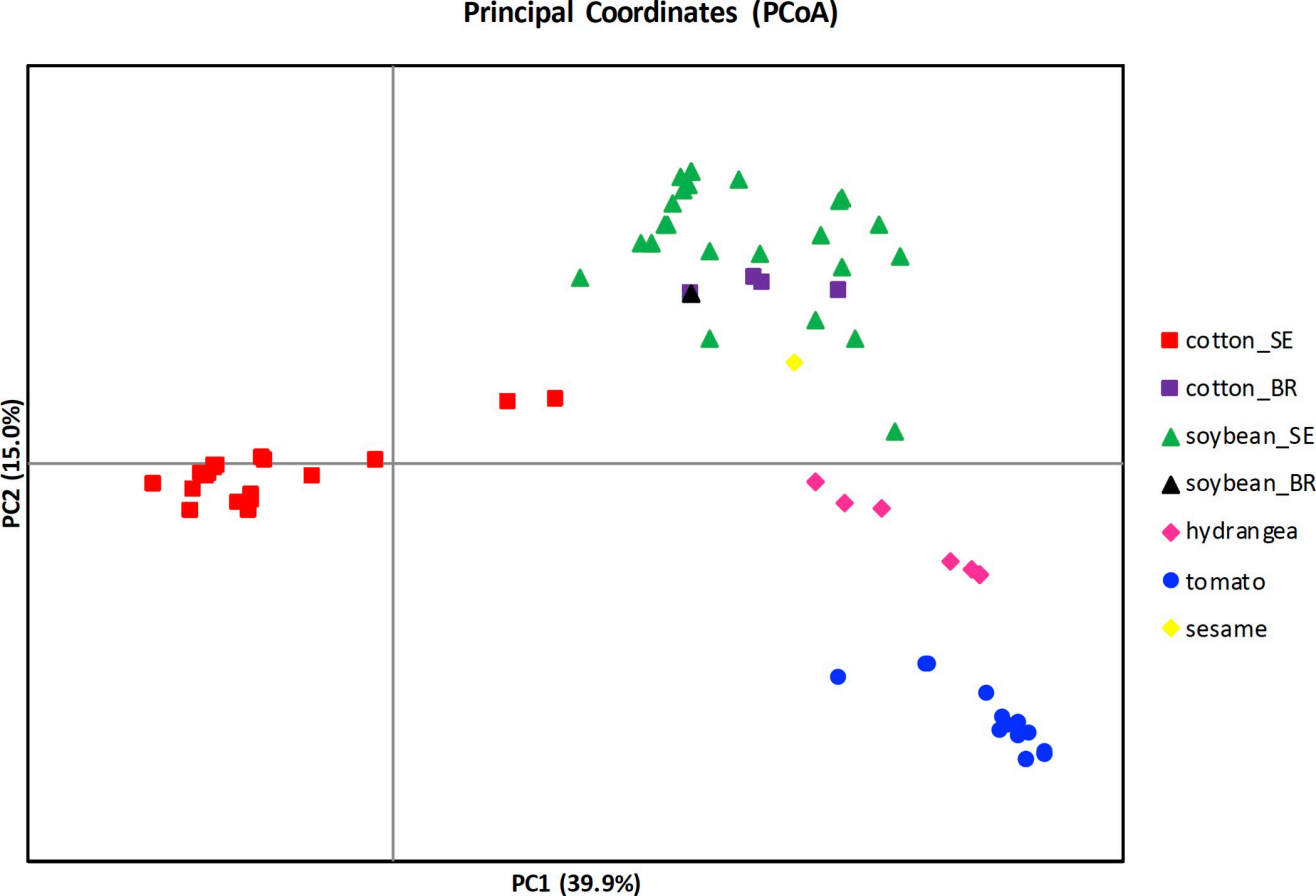
PCoA of 265 *Corynespora cassiicola* isolates based on pairwise genetic distance of multilocus genotypes. The first two principal coordinates are shown. Genotypes from each host are represented by the shape and color shown in the key. Genotypes from cotton and soybean from the southeastern U.S. (SE) or Brazil (BR) are distinguished by color. The tomato population also includes pepper, cucumber, and mandevilla isolates.

The PCoA analysis (Fig 3) showed clustering of *C*. *cassiicola* genotypes from the southeastern U.S. based on host of origin. PC1 and PC2 represent 39.9% and 15.0% of the total variation, respectively. The genotypes of cotton and soybean isolates from Brazil clustered with populations from soybean in the southeastern U.S. Most of the genotypes from cotton in the U.S. cluster tightly together; however, two were midway between most U.S. cotton isolates and the majority of the genotypes from soybean. Based on one-way ANOVA, there were significant differences among populations (cotton, soybean, hydrangea, and the tomato/pepper/cucumber/mandevilla population) in the southeastern U.S. for both PC1 (*p* < 0.001, degrees of freedom = 3, *F* = 2407.9) and PC2 (*p* < 0.001, degrees of freedom = 3, *F* = 1612.2).

**Fig 4:**
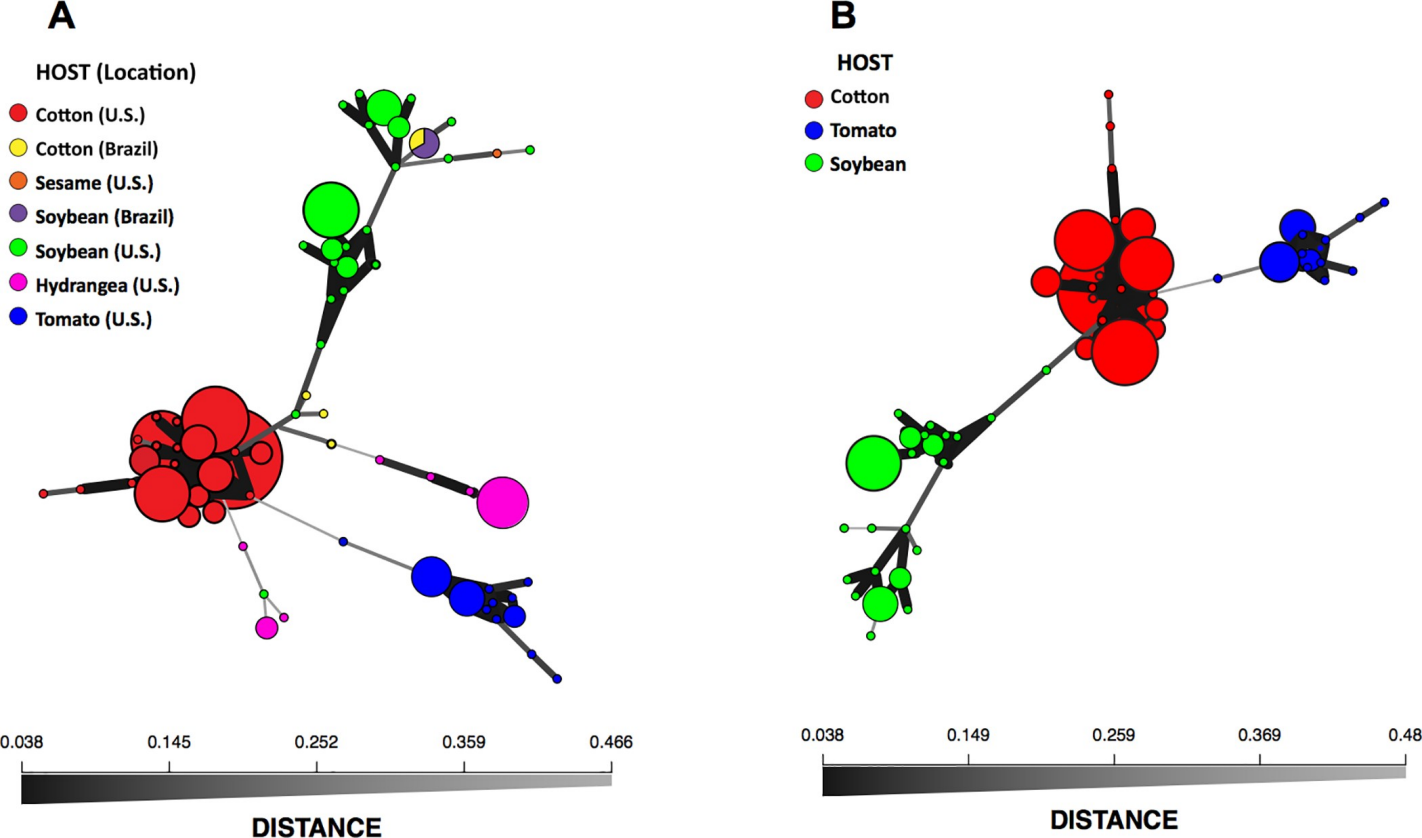
**Minimum spanning networks based on Bruvo’s genetic distance for *Corynespora cassiicola* isolates from: A. cotton, soybean, tomato, hydrangea, cucumber, pepper, mandevilla, and sesame in the southeastern US and cotton and soybean in Brazil; and B. cotton, soybean, tomato, pepper, cucumber, and mandevilla in the southeastern US.** Each node (circle) represents a unique haplotype with the size proportional to the frequency of the haplotype. The color shown for each haplotype represents the host species of origin. Edges (lines) represent minimum genetic distances between haplotypes determined by Prim’s algorithm. Nodes that are more closely related have thicker and darker edges, whereas nodes that are more distantly related have lighter and thinner edges. Cucumber, pepper, and mandevilla belong to the tomato population since they form the same cluster and share haplotypes [21].

The minimum spanning networks for *C*. *cassiicola* based on Bruvo’s genetic distance model (Fig 4A and 4B) show the relationships among haplotypes within and among host populations from the southeastern U.S. and Brazil (Fig 4A) and among the emerging populations from cotton, soybean and tomato in the southeastern U.S. (Fig 4B). Haplotypes from the same host population clustered together with short, thick lines that indicate genetic similarity; however, haplotypes from different hosts were genetically more distantly related as illustrated with thinner, grey lines. Reticulations were not observed among populations in either network, but were observed within the soybean, tomato, and cotton populations.

**Fig 5:**
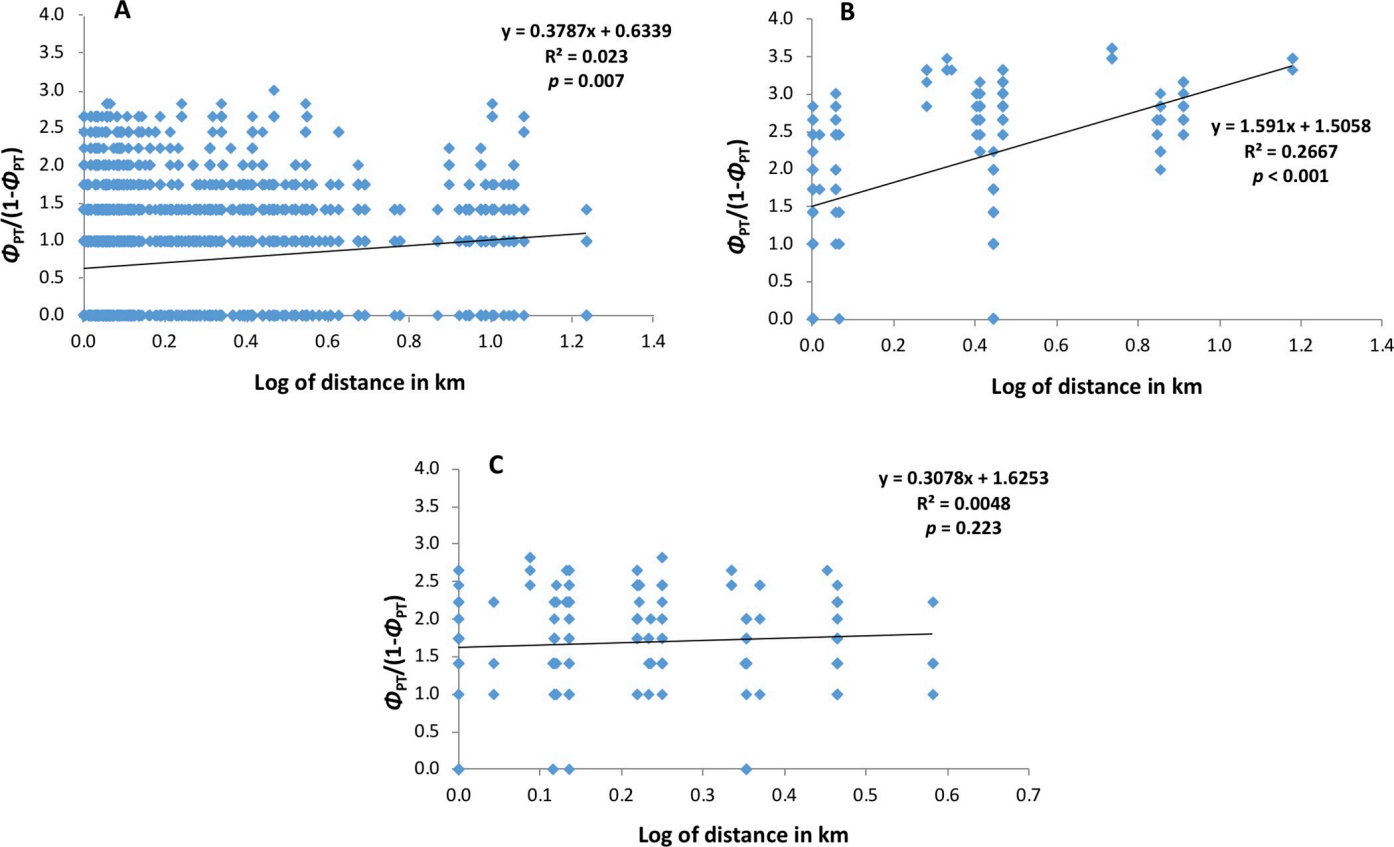
**Regression of pairwise genetic distances and geographic distances of isolates from: A. cotton; B. soybean; and C. tomato in the southeastern US.** The *p*-values are based on 1,000 permutations. The tomato population includes isolates from pepper, cucumber, and mandevilla.

To gain a better understanding on the geographic structure of the target spot epidemics in the southeastern U.S., *Mantel Tests* were conducted to determine correlations between genetic and geographic distances of the *C*. *cassiicola* populations from cotton, soybean, and tomato. Regression analyses on the matrices of pairwise genetic distance and geographic distance revealed positive significant linear correlations for the cotton (*R*^2^ = 0.023, *p* = 0.007) and soybean (*R*^2^ = 0.2667, *p* < 0.001) populations (Fig 5A and 5B) with isolates increasing in genetic distance with increasing geographic distance. However, no significant correlation (*R*^2^ = 0.0048 of *p* = 0.223) was detected for the tomato population (Fig 5C).

## Discussion

To improve our understanding of the genetic diversity, population structure, modes of reproduction, and mechanisms of dispersal of *C*. *cassiicola* causing epidemics in the southeastern U.S., we developed 13 polymorphic microsatellite markers using a next-generation sequencing approach. The markers were informative for genotyping 265 *C*. *cassiicola* isolates from cotton, soybean, tomato, pepper, cucumber, *Mandevilla*, *Hydrangea*, and sesame, that were mostly sampled from southeastern U.S. with a few isolates from cotton and soybean from Brazil (Table 1). The microsatellite markers were highly variable, reproducible and relatively inexpensive, especially when multiplexed, and can be used in future studies to genotype isolates of *C*. *cassiicola* from diverse hosts, geographic ranges, and phylogenetic lineages. There were some issues amplifying some of the loci for the samples from tomato, such as significantly low or no peaks, especially when they were multiplexed. However, we were able to amplify the loci in singleplex reactions. Problems can occur in the multiplexing step, and could be due to various reasons that can affect amplification such as locus-to-locus imbalance [52]. The isolates from tomato belong to a phylogenetic lineage of *C*. *cassiicola* (PL4) that is distantly related to the lineage that most of the other isolates belong (PL1) [21, 22], which could explain the difficulty in amplification. We intentionally used diverse isolates during marker development to ensure they would work for most members of the species. Even though the markers were developed to optimize diversity in cotton isolates they were even more polymorphic for the tomato population.

With the highly variable microsatellite markers we developed, we were able to detect multiple multilocus genotypes within populations of *C*. *cassiicola* from both cotton and tomato (Table 3), which were not detected using a multilocus sequencing approach [21]. Moreover, there was a higher level of genotypic diversity detected within each of the *C*. *cassiicola* populations than expected for a fungus only known to reproduce asexually. Nevertheless, an abundance of overrepresented genotypes were observed supporting high levels of clonality within host-specialized populations (Table 3). Clonal genotypes were found within each of the populations from cotton, soybean, and tomato, but were not found across these host-specialized populations. Similar to *C*. *cassiicola*, other asexually-reproducing fungi have also been reported to have high genotypic diversity within clonal lineages [53, 54]. However, some fungi previously thought to be strictly asexual, have later shown evidence for cryptic sexual reproduction including identification of genetic recombination signatures and/or presence of both mating types [1, 31]. Recombination can be inferred in population genetic analyses when there are high levels of homoplasy [55]. *Sclerotinia sclerotium*, another genetically diverse fungus with a wide host range, showed high levels of homoplasy in combined gene genealogies due to recombination [56]. Homoplasy can also occur within clonal lineages as a result of recurrent and/or reverse mutations [2–3, 57]. Cryptic sexual reproduction within populations or PL of *C*. *cassiicola* cannot be excluded, but populations from different hosts, as well as the different PL, appear to be clonal.

Population structure based on DAPC (Fig 2) and PCoA (Fig 3) revealed genetic clustering of *C*. *cassiicola* populations in the southeastern U.S. based on host species of origin. This was shown previously based on four nuclear loci, but for a very limited sample size for each population [21]. In the present study, we genotyped 265 isolates and found the same host-associated pattern in population structure. Other studies have also shown stronger support of previously identified patterns by increasing sample size and resolution e.g. correlating VCGs and races of *V*. *dahliae* [54, 58]. Among isolates from the southeastern U.S., the genetic clustering by host species was observed irrespective of the geographic origin of the isolates. Moreover, the pathogen populations differed by host even when sampled from the same geographic location, providing further evidence for host specialization in asexual lineages of *C*. *cassiicola* [21, 22, 29–31]. Populations of fungal pathogens exhibiting host preference despite sympatry suggest restricted gene flow due to the populations undergoing host adaptation [59]. In addition, population genetic structure can be attributed to reproductive isolation that is maintaining the genetic differences in these populations. In *Venturia inaequalis*, the apple scab fungus, population genetic structure was based on the host preference of the fungus, specifically whether or not the preferred *Malus* species carried a resistance gene or not [60]. Some fungal pathogens produce host selective toxins or necrotrophic effectors [61–62], as well as possess accessory chromosomes carrying genes for virulence [63], which can facilitate host specialization. Necrotrophic effectors have been identified for some isolates of *C*. *cassiicola* specialized to rubber, yet the genetic basis for specialization in *C*. *cassiicola* remains unclear. Isolates of *C*. *cassiicola* isolates from cotton and soybean in Brazil share the same group and are genetically similar to the isolates from soybean in the southeastern U.S. Isolates that were genetically identical, based on ERIC/REP-PCR and rDNA molecular techniques, were reported to cause target spot outbreaks on cotton and soybean in Brazil [20]. Our results support the finding that soybean and cotton epidemics of target spot in Brazil may be caused by the same populations, which are similar to soybean populations in the U.S., but different from cotton populations in the U.S. Additional isolates need to be assessed to determine whether these genotypes are representative of the overall population causing the epidemics in Brazil. Occurrence of isolates with no distinct genetic structure would suggest free gene flow in these populations. It is unclear why the same population, which is genetically similar to U.S. isolates from soybean, affects both soybean and cotton in Brazil, but the populations from soybean and cotton are specialized in the southeastern U.S.

We detected no spatial genetic structure for the *C*. *cassiicola* population from tomato, pepper, cucumber, and mandevilla (Fig 5C). There was a significant correlation between the genetic and geographic distances of cotton isolates from the southeastern U.S. (Fig 5A); however, the correlation was low. This suggests widespread dispersal of these populations via long distance transport among the regions where they were sampled [22, 35, 60]. The lack of considerable isolation-by-distance or geographic clustering within these populations supports that these are recently introduced or recently evolved strains that spread rapidly throughout the range [64–65]. Long-distance dispersal could be via airborne conidia or infested seed/planting material resulting to human-mediated dispersal; however, neither mode has been studied for this fungus. Contrary to what was observed in cotton and tomato populations in the southeastern U.S., the populations of *C*. *cassiicola* from soybean may have geographic structure and isolation-by-distance (Fig 5B) suggesting limited dispersal in this population. The isolates collected from Tennessee and Arkansas cluster together, and are in a different cluster than those from Georgia. This structure implies older and more established populations of *C*. *cassiicola* on soybean in the southeastern U.S., which is also supported by the higher genetic diversity observed in soybean populations (Table 3, Figs 2–4)[21]. However, despite a higher regression value in comparison to those from cotton, *Mantel* test for *C*. *cassiicola* from soybean still have shown low correlation. Additional studies addressing fine-scale genetic structure of the spatial structure of *C*. *cassiicola* need to be conducted to better understand pathogen aggregation within fields [24], and mechanisms of dispersal.

Determining the genetic diversity and population structure of *C*. *cassiicola* can help in assessing risks for emerging target spot epidemics and in providing insights for the epidemiology of the disease outbreaks. Moreover, understanding the reproductive biology and the mechanism of dispersal of *C*. *cassiicola* can lead to improved management strategies. The mode(s) of reproduction will affect how rapidly populations overcome host resistance or evolve to evade control by fungicides. We now know that target spot epidemics in the southeastern U.S. are caused by clonally reproducing, host-specialized populations that have some genotypic diversity.

## Supporting information

S1 Table Multilocus genotype (MLG) data for all isolates in this study. Isolate name, host, MLG, collection location, and allele size for 13 loci.
(XLSX)
